# Cost-Effectiveness of HIV Testing Referral Strategies among Tuberculosis Patients in India

**DOI:** 10.1371/journal.pone.0012747

**Published:** 2010-09-16

**Authors:** Lauren M. Uhler, Nagalingeswaran Kumarasamy, Kenneth H. Mayer, Anjali Saxena, Elena Losina, Malaisamy Muniyandi, Adam W. Stoler, Zhigang Lu, Rochelle P. Walensky, Timothy P. Flanigan, Melissa A. Bender, Kenneth A. Freedberg, Soumya Swaminathan, for the CEPAC International investigators

**Affiliations:** 1 Division of General Medicine, Massachusetts General Hospital, Boston, Massachusetts, United States of America; 2 Division of Infectious Diseases, Massachusetts General Hospital, Boston, Massachusetts, United States of America; 3 Harvard University Center for AIDS Research (CFAR), Harvard Medical School, Boston, Massachusetts, United States of America; 4 Department of Orthopedic Surgery, Brigham and Women's Hospital, Boston, Massachusetts, United States of America; 5 Division of Infectious Diseases, Department of Medicine, Brigham and Women's Hospital, Boston, Massachusetts, United States of America; 6 Y.R. Gaitonde Centre for AIDS Research and Education, Chennai, India; 7 Miriam Hospital, Brown University, Providence, Rhode Island, United States of America; 8 Tuberculosis Research Centre, Indian Council of Medical Research, Chennai, India; 9 Division of Infectious Disease, New York University School of Medicine, New York, New York, United States of America; McGill University, Canada

## Abstract

**Background:**

Indian guidelines recommend routine referral for HIV testing of all tuberculosis (TB) patients in the nine states with the highest HIV prevalence, and selective referral for testing elsewhere. We assessed the clinical impact and cost-effectiveness of alternative HIV testing referral strategies among TB patients in India.

**Methods and Findings:**

We utilized a computer model of HIV and TB disease to project outcomes for patients with active TB in India. We compared life expectancy, cost, and cost-effectiveness for three HIV testing referral strategies: 1) selective referral for HIV testing of those with increased HIV risk, 2) routine referral of patients in the nine highest HIV prevalence states with selective referral elsewhere (current standard), and 3) routine referral of all patients for HIV testing. TB-related data were from the World Health Organization. HIV prevalence among TB patients was 9.0% in the highest prevalence states, 2.9% in the other states, and 4.9% overall. The selective referral strategy, beginning from age 33.50 years, had a projected discounted life expectancy of 16.88 years and a mean lifetime HIV/TB treatment cost of US$100. The current standard increased mean life expectancy to 16.90 years with additional per-person cost of US$10; the incremental cost-effectiveness ratio was US$650/year of life saved (YLS) compared to selective referral. Routine referral of all patients for HIV testing increased life expectancy to 16.91 years, with an incremental cost-effectiveness ratio of US$730/YLS compared to the current standard. For HIV-infected patients cured of TB, receiving antiretroviral therapy increased survival from 4.71 to 13.87 years. Results were most sensitive to the HIV prevalence and the cost of second-line antiretroviral therapy.

**Conclusions:**

Referral of all patients with active TB in India for HIV testing will be both effective and cost-effective. While effective implementation of this strategy would require investment, routine, voluntary HIV testing of TB patients in India should be recommended.

## Introduction

India accounts for one fifth of the global burden of tuberculosis (TB), with 1.8 million new cases of active TB each year—more new cases than any other country [1]. The Revised National Tuberculosis Control Programme (RNTCP) in India reports that two in five Indians are infected with latent TB [2]. India also has a substantial burden of HIV; recent estimates from the World Health Organization (WHO) and India's National AIDS Control Organization (NACO) report 2.5 million people living with HIV in India (an overall population HIV prevalence of 0.36%) [2], [3]. HIV co-infection substantially increases the risk of progression from latent TB infection to active TB, and TB is the leading cause of mortality in HIV-infected persons in India [1], [4]. However, recent studies have shown substantial improvement in CD4 counts and decreased mortality among HIV-infected patients who received ART during TB treatment compared to HIV-infected patients without access to antiretrovirals [5], [6].

The RNTCP and NACO addressed the intersection of TB and HIV disease by establishing cross-referral mechanisms between facilities providing TB services and HIV integrated counseling and testing centers [7]. NACO guidelines recommend that all patients with active TB and HIV risk factors be referred for HIV counseling and testing [7]. Yet current estimates show that less than 6% of TB patients are tested for HIV infection [8]. In 2007, the Indian government reported that over 12% of the 77,000 TB patients referred for HIV testing were diagnosed with HIV [9]. National TB/HIV policy in India is evolving. In 2007, NACO and the Central TB Division established the first National Framework of Joint TB/HIV Collaborative Activities, expanding basic TB/HIV activities to all states [7]. In October 2008, guidelines were changed to implement an Intensified TB/HIV Package in the nine states with the highest HIV prevalence, including referral to HIV counseling and testing sites for HIV tests free of charge for all TB patients, with continued selective referral in the other 26 states [2], [10]. The National Framework for Joint TB/HIV Collaborative Activities was further revised in 2009 to establish uniform guidelines at counseling and testing centers and ART centers nationwide, to standardize monitoring and evaluation, and to expand the Intensified TB/HIV Package to all states by 2012 [11]. The TB/HIV Collaborative Activities also include routine TB screening for patients attending HIV testing centers and ART centers; however, the current analysis addresses HIV testing referral for TB patients. Our objective was to project the clinical and economic outcomes of alternative referral strategies for HIV testing among TB patients in India.

## Methods

### Analytic Overview

We use the Cost-Effectiveness of Preventing AIDS Complications (CEPAC) International model, a state-transition simulation model of HIV and TB disease in resource-limited settings, to project the life expectancy, cost, and cost-effectiveness of HIV testing for patients with active TB in India. Details of the model are published elsewhere [12], [13], [14], [15], [16], [17]. Input parameters for the model include data on both TB and HIV natural history, treatment efficacy, and costs of care from India; model outputs include projected per-person life expectancy, lifetime cost, and cost-effectiveness. Life expectancy and cost are discounted at 3% per year [18].

We estimate life expectancy and costs for three different HIV testing referral strategies for TB patients: 1) selective referral of patients considered to be at high risk for HIV, hereafter referred to as “selective referral,” 2) routine referral of patients in the nine highest HIV prevalence states with selective referral in the other 26 states (the “current standard”), and 3) routine referral of all patients for HIV testing, hereafter “routine referral.” Patients at “high-risk” for HIV—those targeted for HIV testing under the “selective referral” strategy—include those who report a history of high-risk behavior, who have a history of sexually transmitted infection, and/or who have signs and symptoms suggestive of HIV-related opportunistic infections [7]. For each HIV testing referral strategy, patients who are referred for HIV testing, offered a test, and accept testing, receive one rapid HIV test; those with reactive tests receive a confirmatory rapid test. For each strategy, the overall probability that a patient is referred for HIV testing and receives the test is referred to as the “probability of offer/accept” [9], [19]. There are three HIV testing-related outcomes: 1) HIV-negative, 2) HIV-infected, but not tested or linked to HIV care and therefore not treated, and 3) HIV-infected, tested, linked to care, and treated (Figure S1). Patients with HIV infection who are not tested initially enter HIV care later in the course of disease, upon the occurrence of a severe opportunistic infection (excluding bacterial infections and recurrent TB). HIV-infected patients who are tested and linked to care, but not yet eligible for antiretroviral therapy (CD4 count>350/µl), are monitored with regular clinic visits every 3 months and receive treatment for acute opportunistic infections. They also have CD4 counts every 6 months, and are initiated on antiretroviral therapy (ART) once their CD4 count falls below 350/µl.

We were guided by the Commission on Macroeconomics and Health, sponsored by the WHO, in determining if a particular HIV testing strategy is considered “cost-effective.” Strategies with incremental cost-effectiveness ratios <3 times the *per capita* Gross Domestic Product (GDP) in India (3x *per capita* GDP = $3,050) are considered “cost-effective,” while strategies with incremental cost-effectiveness ratios <1 times the *per capita* GDP (1x *per capita* GDP = $1,015) are considered “very cost-effective” [20], [21].

### Tuberculosis Disease States

The CEPAC International model incorporates detailed data with respect to both TB and TB/HIV co-infection. There are five mutually exclusive TB health states considered: no TB exposure, latent TB infection, active TB disease without treatment, active TB disease with treatment, and history of active TB. Patients transition among health states if they experience a primary latent or active TB infection, re-infection, relapse of an active infection, or spontaneous resolution, or if they complete treatment successfully or fail treatment. The four TB outcomes considered are: cured, failed TB treatment, defaulted (TB treatment interrupted for at least two consecutive months), or died.

### HIV Disease and Treatment

The natural history of HIV disease is determined by CD4 count decline, the rate of which depends on HIV RNA level [22]. HIV morbidity and mortality are CD4 count-dependent, with higher morbidity and mortality at lower CD4 counts [23]. ART reduces HIV RNA levels, increases CD4 counts, and thus decreases HIV-related morbidity and mortality [36]. Data on virologic efficacy and CD4 count increases due to ART are from a published trial [24]. ART regimens and treatment policies follow recommendations from NACO and the WHO [25], [26]. First-line ART is a non-nucleoside reverse transcriptase inhibitor (NNRTI)-based regimen consisting of nevirapine and two nucleoside reverse transcriptase inhibitors (NRTIs), usually stavudine and lamivudine. HIV co-infected patients in care have CD4 count tests every 6 months and are treated with co-trimoxazole and two sequential ART regimens once their CD4 count falls to <350 cells/µl, per NACO guidelines [25], [27]. All TB-related outcomes are worse for HIV-infected patients than for HIV-negative patients and for HIV-infected patients who do not receive ART compared to those on ART [28]. All HIV-infected patients are subject to a monthly risk of mortality from both TB and HIV.

### Model Input Data

#### Cohort Characteristics

Baseline characteristics reflect the composition of patients enrolled in a study of short-course anti-TB treatment at the Tuberculosis Research Centre in Chennai, India [29]. Mean age at initial HIV test is 33.50 years (SD 7.20 years), and 83% are male (Table 1). The overall prevalence of HIV disease in TB patients is 4.9% and among those with TB/HIV co-infection, mean CD4 count is 169/µl (SD 126/µl) [29], [30]. Additional cohort characteristics are shown in Table S1.

**Table 1:** Baseline cohort characteristics, TB treatment outcomes, and model inputs for an analysis of HIV testing for TB patients in India.

Variable	Base case input	Range used in sensitivity analyses	Reference
**Cohort Characteristics**			
Age, years (SD)	33.5 (7.2)	25.0–45.0	[29]
Male/Female (%)	83/17		[29]
HIV prevalence among TB patients (%)	4.9	0.0–10.0	[30]
Mean CD4 count at HIV diagnosis, cells/µl (SD)	169 (126)	85–335	[29]
**TB Treatment Outcome (at 1 year from TB diagnosis)** ‡			
HIV-negative population, % within each subgroup			[31]
Cured	83	73–93	
Failed TB treatment	3	1–6	
Defaulted on TB treatment	8	4–12	
Died	6	3–9	
HIV-infected population, % within each subgroup			[31]
Cured	61	51–71	
Failed TB treatment	3	1–6	
Defaulted on TB treatment	14	9–19	
Died	22	17–27	

SD: Standard deviation; TB: Tuberculosis.

‡ Cured: smear-negative in the last month of treatment; Failed: Remained smear-positive at month 5 or later during TB treatment; Defaulted: TB treatment was interrupted for at least 2 consecutive months; Died: Died from any cause during TB treatment [31].

#### TB-related Data

The proportion of each TB treatment outcome in the HIV-negative and HIV-infected patients is from the WHO [31]. For HIV-negative patients at six months, 83% are cured of TB, 3% fail TB treatment, 8% default from TB treatment, and 6% die (Table 1) [31]. Of the TB patients with HIV co-infection, at six months 61% are cured of TB, 3% fail TB treatment, 14% default from TB treatment, and 22% die [31]. Mean survival and cost of each TB treatment outcome are weighted according to the proportion of those outcomes as reported by WHO to determine the overall survival and cost for the simulated cohort (Table 1; Tables S2, S3, S4). Mortality rates for each TB outcome are midpoint assumptions on survival for HIV-negative TB patients in India (Table 2).

**Table 2:** TB mortality and ART efficacy model inputs for an analysis of HIV testing for TB patients in India.

Variable	Base case input	Range used in sensitivity analyses	Reference
**Mortality from 1–3 years following TB diagnosis, according to HIV serostatus and TB treatment outcome subgroup (%)**			
HIV-negative			
Cured	6	3–12	[59]
Failed TB treatment	34	17–68	[59]
Defaulted on TB treatment	42	21–84	[59]
HIV-infected, untreated for HIV			
Cured	37	19–74	[29]
Failed TB treatment	68	34–95	Assumption*
Defaulted on TB treatment	84	42–95	Assumption*
HIV-infected, treated (ART)			
Cured	10	5–20	Assumption*
Failed TB treatment	50	25–75	Assumption*
Defaulted on TB treatment	84	42–95	Assumption*
**Efficacy of antiretroviral therapy**			
HIV RNA suppression at 24 weeks (%)	73	60–90	[24]
CD4 count increase at 24 weeks (cells/µl)	148	100–200	[32]

ART: antiretroviral therapy; TB: Tuberculosis.

*Assumed 67% of mortality occurred in months 12–18 and 33% occurred in months 19–36. See Text S1 for further details on extended mortality.

#### HIV Disease and Treatment

Data on HIV natural history are from the Y.R. Gaitonde Center for AIDS Research and Education (YRG CARE) in Chennai, India [23]. The efficacy of a first-line NNRTI-based ART regimen is 73% virologic suppression (HIV RNA <50 copies/ml) at 24 weeks with a mean CD4 increase of 148/µl at 24 weeks (Table 2) [24], [32]. Second-line ART, consisting of ritonavir-boosted lopinavir, zidovudine and emtricitabine, was assumed to have similar efficacy as first-line ART. Additional details on the clinical input data have been published elsewhere [12], [13], [33].

#### HIV Testing Offer/Acceptance and Outcomes

Selective referral (Strategy 1) has a test offer/accept probability of 5.2% and detects 13.2% of those with HIV (Table 3) [9]. The current standard (routine referral in the nine highest HIV prevalence states, with selective referral elsewhere; Strategy 2), has a test offer/accept probability of 22.7% and detects 44.7% of those with HIV. Routine referral for all TB patients (Strategy 3) has a test offer/accept probability of 66.2% and detects 66.2% of those with HIV [9], [19]. The Technical Appendix (Text S1) contains a detailed description of calculations for HIV test offer/accept and the proportion of HIV among TB patients detected. Of ART-eligible patients newly identified as HIV-infected under Strategy 3, 26.0% link to HIV care, defined as starting ART if eligible [19]. We assumed the same linkage to care rates for Strategies 1 and 2, but varied this assumption in sensitivity analysis.

**Table 3:** Rates of HIV test offer and acceptance, proportion of HIV/TB co-infection detected, and linkage to HIV care, stratified by referral strategy, for TB patients in India.

Parameter	Strategy 1: Selective referral		Strategy 2: Routine referral in 9 states with high HIV prevalence		Strategy 3: Routine referral in all states	
	Data	Source	Data	Source	Data	Source
Total # of TB patients registered for treatment	1,475,587	*RNTCP* [*9*]	1,475,587	*RNTCP* [*9*]	1,475,587	*RNTCP* [*9*]
Probability of HIV test offer/accept	5.2%	*RNTCP* [*9*]	22.7%	*RNTCP* [*9*] * Vijay, et al.* [*19*]	66.2%	*Vijay, et al.* [*19*]
# of TB patients tested for HIV	77,000	*RNTCP* [*9*]	335,460	Calculation¥	976,839	Calculation¥
HIV prevalence in TB patients tested for HIV	12.3%	*RNTCP* [*9*]	9.0% (high prev. states), 12.3% (other states)	Calculation¥	4.85%	Calculation¥
Actual HIV prevalence in TB patient population	4.85% across all TB patients	*RNTCP* [*2*]	9.0%§ (high prev. states), 2.9%‡ (other states)	*Raizada et al.* [*10*]	4.85% across all TB patients	*RNTCP* [*2*]
Proportion of HIV-TB co-infection detected	13.2%	Calculation¥	44.7%	Calculation¥	66.2%	Calculation¥
Probability of linkage to HIV care*	26%	*Vijay, et al.* [*19*]	26%	*Vijay, et al.* [*19*]	26%	*Vijay, et al.* [*19*]

RNTCP: Revised National Tuberculosis Control Programme [9].

§ Average HIV seroprevalence among TB patients in the districts in the 4 high prevalence states included in Raizada 2008: Andhra Pradesh, Karnataka, Maharashtra, Tamil Nadu [10].

‡ Average HIV seroprevalence among TB patients in all districts in the 4 low prevalence states included in Raizada 2008: Junagadh and Vadodara, Gujarat; Thrissur, Kerala; Jodhpur, Rajasthan; Koch Bihar and Uttar Dinajpur, West Bengal [10].

¥ See Text S1 for a detailed description of calculations.

*Linkage to HIV care is defined as the percent of TB patients starting ART for those determined to be ART-eligible. Probability for Strategies 1 and 2 assumed similar to Strategy 3 [19].

#### Costs of Testing and Care

Resource utilization data are from the YRG CARE database, as well as from a daily cost analysis of YRG CARE conducted by Family Health International [34]. Input parameters include the number of inpatient and outpatient days associated with routine HIV care, acute HIV-associated opportunistic infections, and death. ART drug costs are from India's National AIDS Control Organisation [35]. Annual per-person costs of first- and second-line ART are $108 and $690, and the annual cost of co-trimoxazole prophylaxis is $4 (Table 4) [35], [36]. The cost of TB treatment, consisting of ethambutol, isoniazid, rifampicin and pyrazinamide three times weekly for two months, followed by rifampicin and isoniazid three times weekly for four months, is $34 [37]. The cost of an HIV test is $3, including the salary of the counselor but excluding infrastructure costs related to scale-up of HIV testing [38]. All costs are standardized to 2008 US dollars using India's 2008 GDP deflator [21].

**Table 4:** Cost inputs for an analysis of HIV testing for TB patients in India.

Variable	Base case input	Range used in sensitivity analyses	Reference
**Costs (2008 US$)**			
First-line ART, yearly§	108	36–180	[35]
Second-line ART, yearly§	690	300–1,200	[35]
Co-trimoxazole prophylaxis, yearly	4	4–11	[36]
TB treatment¥	34	10–100	[37]
HIV test cost	3	3–30	

ART: antiretroviral therapy; TB: Tuberculosis.

§ First-line ART: non-nucleoside reverse transcriptase inhibitor-based regimen; Second-line ART: protease inhibitor-based regimen (See Methods for details).

¥ Cost of TB treatment includes personnel costs, cost of the drug regimen, and cost of a sputum smear and chest x-ray [37].

#### Sensitivity Analyses

Univariate sensitivity analyses are performed on key model parameters to assess how changes in these parameters affect the results. We also performed two-way sensitivity analyses on parameters with the greatest impact on the results. HIV-related parameters examined include HIV prevalence, percent of patients tested for HIV, linkage to HIV care after a positive HIV test, and HIV test cost. ART parameters examined in sensitivity analyses include efficacy, availability, and cost of both first- and second-line ART, and TB parameters examined include TB treatment outcomes, rates of TB mortality and cost of TB treatment.

## Results

### Survival and Cost for HIV-Negative and HIV-Infected Patients

Among HIV-negative patients, those who were cured of TB, failed TB treatment, or defaulted, had mean undiscounted life expectancies, beginning at age 33.50, ranging from 33.89 years down to 18.38 years (Table 5). Mean per person lifetime TB-related costs ranged from $20–$75.

**Table 5:** Life expectancy and cost outcomes for groups of TB patients in India, stratified by HIV infection and treatment status.

TB outcome according to HIV serostatus	Outcome frequency (%)	Undiscounted life expectancy (years)	Lifetime cost (US$)^1^
HIV-negative			
Cured	83	33.89	75
Failed TB treatment	3	21.60	45
Defaulted on TB treatment	8	18.38	20
Died	6	0.37	40
HIV-infected, untreated for HIV¥			
Cured	61	4.71	1,990
Failed TB treatment	3	2.57	1,035
Defaulted on TB treatment	14	1.33	440
Died	22	0.35	55
HIV-infected, treated with antiretroviral therapy*			
Cured	61	13.87	7,840
Failed TB treatment	3	7.05	3,875
Defaulted on TB treatment	14	2.03	985
Died	22	0.36	135

1 Costs include $12 end of life care cost and opportunistic infection treatment costs, but do not include $3 HIV test cost. Costs are in 2008 US$.

¥ HIV-infected, untreated for HIV still receive treatment for acute opportunistic infections.

*Per Indian guidelines [25].

TB patients who were HIV-infected, but not tested or treated for HIV, and who survived 6 months with TB, had projected undiscounted mean life expectancies ranging from 1.33 years to 4.71 years, and costs ranging from $440-$1,990, depending on their TB outcomes. For those tested and treated for HIV, both mean undiscounted life expectancy and lifetime costs increased substantially compared to those with untreated HIV. HIV-infected patients on ART who survived 6 months with TB had projected life expectancies ranging from 2.03 years to 13.87 years, depending on TB outcomes. Lifetime costs for those tested and treated for HIV and who survived 6 months with TB, ranged from $985–$7,840. Across all TB outcomes, HIV-infected patients treated for HIV had a projected mean life expectancy of 9.04 years, compared to 3.21 years for those HIV-infected but not treated for HIV.

### Base Case Analysis

The mean life expectancy and costs for each of the 12 subgroups of TB and HIV patients were weighted by the frequency of their occurrence to determine the overall projected mean life expectancy and cost for each HIV testing referral strategy (Tables S2, S3, S4). For Strategy 1 (selective referral), discounted mean life expectancy was projected to be 16.88 years with a mean discounted lifetime cost of $100 (Table 6). Strategy 2 increased survival by 0.02 years at an additional cost of $10, for an incremental cost-effectiveness ratio of $650/year of life saved (YLS) compared to Strategy 1. Strategy 3 (routine referral), further increased discounted life expectancy by 0.01 years and costs by $10 compared to Strategy 2, with an incremental cost-effectiveness ratio of $730/YLS.

**Table 6:** Incremental cost-effectiveness of alternative HIV testing strategies for TB patients in India.

Strategy	Discounted mean per-person life expectancy, years (undiscounted)*	Discounted mean per-person lifetime costs (undiscounted)**	Cost-effectiveness ratio, $/YLS
Strategy 1: Selective referral of high-risk patients	16.88 (28.90)	100 (135)	—
Strategy 2: HIV testing for all TB patients in the nine highest HIV prevalence states	16.90 (28.92)	110 (150)	650
Strategy 3: Routine referral for HIV testing for all TB patients	16.91 (28.93)	120 (160)	730

YLS: Year of life saved.

*Life expectancy beginning at age 33.50 (see Methods).

**Costs are in 2008 US$.

### Sensitivity Analyses

**Figure 1:**
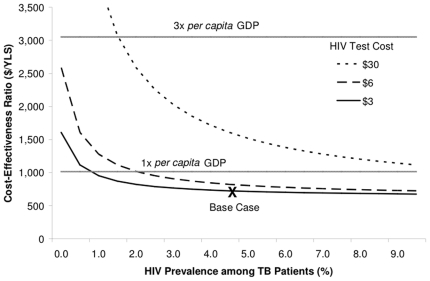
Sensitivity analysis on HIV prevalence among TB patients. The cost-effectiveness of routine referral for HIV testing for all TB patients (Strategy 3) compared to the current standard of referral for HIV testing for TB patients in the nine states with the highest HIV prevalence and selective referral elsewhere (Strategy 2), as a function of HIV prevalence and HIV test cost. The 3x and 1x *per capita* GDP for India represent thresholds for “cost-effective” and “very cost-effective” health care interventions, as recommended by the WHO (See Methods). GDP: Gross Domestic Product; YLS: Year of life saved.

Sensitivity analyses showed that results were most sensitive to the prevalence of HIV in patients receiving TB treatment. However, until the prevalence dropped below 0.5%, substantially below the reported HIV prevalence among TB patients in any setting in India, the cost-effectiveness ratio for Strategy 3 (routine referral) remained less than 3x the *per capita* GDP in India, $3,050/YLS (Figure 1). In multivariate sensitivity analysis, we varied HIV test cost simultaneously with HIV prevalence; even if the HIV test cost was increased 10-fold, to $30/test, Strategy 3 still had a cost-effectiveness ratio below $3,050/YLS, unless the HIV prevalence among TB patients was also less than 2.5% (Figure 1, dotted line).

Although TB treatment outcomes affected projected survival substantially, they did not have an important effect on cost-effectiveness results. If the proportion of patients cured of TB increased an absolute 10%—to 93% for HIV-negative patients and to 71% for HIV-infected patients—and the proportion of patients defaulting, failing treatment, or dying decreased, the projected life expectancy for Strategy 2 increased from 16.90 years to 18.07 years, while the life expectancy for Strategy 3 increased from 16.91 years to 18.08 years. The cost-effectiveness of Strategy 3 compared to Strategy 2 decreased to $720/YLS. If the proportion of patients cured of TB decreased an absolute 10%—to 73% for HIV-negative patients and to 51% for HIV-infected patients—and the proportion of other TB outcomes increased, the projected life expectancy decreased to 15.83 years for Strategy 2 and 15.84 years for Strategy 3, while the cost-effectiveness ratio of Strategy 3 compared to Strategy 2 increased to $760/YLS. We also decreased the rates of death to 13% for HIV-infected patients not receiving ART and 8% for HIV-infected patients receiving ART to match the results of a recent meta-analysis by Khan *et al.* on TB treatment among HIV-infected patients [39]. This change did not affect the policy results.

The cost of second-line ART had an important impact on the cost-effectiveness results. As costs decreased from the baseline of $690/year, HIV testing in all strategies became more cost-effective. Decreasing the cost of second-line ART to $300/year, the cost-effectiveness ratio of Strategy 3 compared to Strategy 2 improved from $730/YLS to $350/YLS. Increasing the cost of second-line ART to $1,200/year increased the cost-effectiveness ratio of Strategy 3 to $950/YLS. If second-line ART was not available, then routine referral for HIV testing led to lower life expectancy, but its cost-effectiveness ratio was $430/YLS, still less than 1x the *per capita* GDP in India.

Results were not sensitive to initial age, CD4 count at diagnosis, TB mortality rates, rates of virologic suppression on first- or second-line ART, cost of TB treatment, or HIV test cost. The rates of test offer/accept and linkage to care also had little effect on the results. Higher rates of test offer/accept and linkage to care improved survival and increased costs, but had little impact on cost-effectiveness.

## Discussion

The Revised National TB Control Programme in India currently recommends referral for voluntary HIV counseling and testing of all TB patients in the nine highest HIV prevalence states, with risk-based referral elsewhere [7]. While full implementation of this policy would make major progress in HIV case identification, it is still a program of selective referral and likely misses many additional patients who could also benefit from HIV testing and ART. Using a detailed simulation model of HIV and TB disease, we found that a strategy of routine referral for HIV testing in all TB patients in India compared to HIV testing referral in only the nine highest HIV prevalence states (the current standard) may lead to substantial individual survival benefits. HIV-infected TB patients who were treated for HIV disease had an undiscounted per-person mean life expectancy increase of 5.83 years compared to those not treated for HIV, regardless of TB outcome. For those who remained in TB care, and were cured of TB, referral for HIV testing and linkage to care had the greatest impact on survival. Patients cured of TB who linked to care and received ART had an undiscounted mean life expectancy of 13.87 years, or an increase of 9.16 years over those cured of TB but not treated for HIV.

Referral for HIV testing of all TB patients in India compared to HIV testing referral in only the nine highest HIV prevalence states had an incremental cost-effectiveness ratio of $730/YLS. Routine referral for HIV testing of all TB patients in India would be considered very cost-effective (cost-effectiveness ratio <1x *per capita* GDP) [20]. In fact, NACO and the RNTCP have recently proposed routine HIV counseling and testing for all TB patients nationwide by 2012. The current study supports this policy decision and highlights that the policy will be very cost-effective. Our findings also support the current WHO recommendation of routine referral for HIV testing for TB patients in all settings [40]. Our analysis examined HIV testing referral for TB patients, so an analysis of screening HIV-infected patients for TB or of cross-referral services would provide additional important policy information.

In 2009, NACO and the RNTCP revised the “National Framework for Joint TB/HIV Collaborative Activities” to strengthen NACO and RNTCP coordination, as well as coordination between HIV counseling and testing centers and ART centers [11]. The revised framework also establishes mechanisms for better reporting, monitoring, and evaluation of the programs, and provides additional training on TB and HIV for staff. A commitment to screen for HIV infection must be coupled with a commitment to HIV care. Although ART scale-up has led to 272 treatment centers and 10 Regional Centers of Excellence nationally, with integrated counseling and testing services available in every district in India, whether they would be able to accommodate the increased patient burden resulting from routine HIV testing referral is unclear [41]. Successfully implementing a routine HIV testing referral program for the approximately 1.8 million people starting TB treatment each year in India will require a major commitment. One recent pilot study of routine referral for HIV testing in India found that patients identified as HIV-infected had low rates of ART initiation, even when they met immunological starting criteria [19]. Further expansion and decentralization of HIV diagnostic and treatment services is underway to prepare for the expanded implementation of the Intensified TB/HIV Package and to provide access to HIV services closer to patients' homes [11], [19].

Routine testing can detect HIV infection among individuals in an earlier disease stage, before their CD4 count falls to low levels associated with high morbidity and mortality. Currently, 85% of people living with HIV and registered for ART in India registered when their CD4 count was already <250/µl [42]. With earlier HIV diagnosis, patients can initiate ART at higher CD4 counts, and prevent much of the morbidity and mortality that occurs in lower CD4 strata [17]. TB morbidity and mortality in India is a major driver of global TB outcomes, given the size of India's population. The Millennium Development Goals include halving TB prevalence and death rates between 1990 and 2015. Reducing mortality among HIV-infected TB patients has been identified as an important target for India to reach the Millennium Development Goals; identifying and treating HIV-infected TB patients with ART could contribute substantially toward reaching this target [43]. ART has also been demonstrated to reduce the risk of TB for HIV-infected patients, by lowering HIV RNA levels and increasing CD4 counts [44], [45]. However, in order for the clinical benefits and cost-effectiveness of routine HIV testing to be realized, linkage to HIV care and treatment must be maximized [46]. One potential strategy to maximize patient retention between the TB center and HIV counseling and testing center would be to integrate HIV testing and treatment into TB services.

The results of this analysis were robust across a wide range of sensitivity analyses. Results depended on HIV prevalence, but the policy implications did not change unless the prevalence was substantially below that reported anywhere in India [10]. Neither outcomes nor cost from TB or HIV disease, including TB-related mortality rates, had a major impact on the results. Even decentralized HIV testing in India, in areas with relatively low HIV prevalence, will be both effective and cost-effective. Since HIV is common in patients with TB, routine referral for voluntary HIV testing of all TB patients will lead to substantial survival benefits, since effective HIV treatment is now widely available in India [24]. Even if the likelihood of patients linking to HIV care was decreased by 40% with routine referral, HIV testing of all patients remained very cost-effective. Although lower linkage rates did adversely affect survival, the impact of linkage on cost-effectiveness is minimal, because as fewer people link to HIV care, both costs and survival decrease. The cost of second-line ART had an impact on the cost-effectiveness of HIV testing, since the cost-effectiveness is highly dependent on the lifetime cost of HIV care, which in turn depends crucially on the cost of second-line therapy. If second-line ART was not available, the survival benefits of HIV testing were less, but the cost-effectiveness results were robust.

HIV testing of TB patients may also confer further benefits in India through its impact on HIV prevention. Although not directly evaluated in the current study, many studies from resource-limited settings have shown that patients testing positive for HIV significantly decrease high-risk behaviors [47], [48], [49], [50], [51]. The impact of testing negative for HIV on risk-taking behavior is less clear [49]. However, evidence suggests that HIV testing and referral programs will improve HIV prevention, with benefits extending beyond the individual survival benefits highlighted in the current analysis.

The other highest TB burden countries in the world—China, Indonesia, Nigeria, and South Africa—had estimated HIV prevalence in incident TB cases for 2008 ranging from 2.8% (Indonesia) to 71% (South Africa). However, only 22% of TB patients worldwide were tested for HIV in 2008 [52]. Because providing ART is essential to decreasing mortality from TB for HIV-infected persons, detecting HIV earlier, through routine referral for HIV testing for all patients receiving TB treatment, will substantially increase their survival, and likely be cost-effective in other high TB burden countries. HIV testing and earlier initiation of ART may also have prevention benefits by decreasing HIV transmission [50], [51], [53].

There are several limitations to this study. First, data on long-term outcomes for HIV-infected patients cured from TB disease remain scarce. We used the best available data and conducted both univariate and multivariate sensitivity analyses to test the robustness of the results given this uncertainty. While probabilistic sensitivity analysis to simultaneously address uncertainty among all input parameters would be additionally informative, this was not feasible in this Monte Carlo microsimulation. We also assumed in the base case that all patients have an equal probability of linking to HIV care, regardless of disease stage. We did not include the additional benefits of decreasing both HIV transmission and TB transmission by identifying, counseling, and treating HIV disease in TB patients [48], [50], [53], [54], [55]. Including these benefits would render routine HIV testing even more cost-effective [53]. We also did not include disability-adjusted life years (DALYs) in the analysis. Even for those who do not die from TB, the disease may result in substantial disability [56]. Thus, if DALYs were included in the analysis, routine HIV testing might be even more cost-effective. We also used HIV natural history data from a private clinic in Chennai rather than from government facilities. However, since the main determinant of the cost-effectiveness results is HIV prevalence, these results are likely generalizable to other regions of India. While the new WHO recommendations include ART for any HIV-infected patient with TB, regardless of CD4 count, in this analysis only patients with CD4<350/µl were treated with ART [57]. Since all patients in this analysis had active TB, we did not include the new WHO recommendation for isoniazid preventive therapy in patients with HIV in whom TB disease is excluded [58].

Among people with HIV infection in India, TB is the most common opportunistic infection and cause of death [1], [27]. However, the majority of TB patients are not tested for HIV. Our findings suggest that in a country with low overall HIV prevalence, routine voluntary HIV testing of all TB patients would be both clinically effective and very cost-effective. The intersection of the dual epidemics of HIV and TB requires effective HIV and TB case detection and linkage to care to ensure the best possible outcomes for co-infected individuals. Routine voluntary HIV testing for all TB patients in India should be widely implemented.

## Supporting Information

Text S1 Technical Appendix.
(0.05 MB DOC)

Table S1 Baseline cohort characteristics and model inputs for an analysis of HIV testing for TB patients in India.
(0.10 MB DOC)

Table S2 Weighted cost and life expectancy by TB outcomes.
(0.05 MB DOC)

Table S3 Weighted cost and life expectancy by TB outcomes (continued).
(0.05 MB DOC)

Table S4 Weighted cost and life expectancy by TB outcomes (continued).
(0.03 MB DOC)

Figure S1 Twelve patient subgroups in a model of HIV testing among TB patients in India. Grey shading indicates those patients tested and treated for HIV infection.
(2.52 MB TIF)
