# Supplementary material for: Cost-Effectiveness of HIV Testing Referral Strategies among Tuberculosis Patients in India
Source: PLoS One. 2010 Sep 16;5(9):e12747. doi: 10.1371/journal.pone.0012747 (PMC2940842; doi:10.1371/journal.pone.0012747)
Supplement: Text S1 — Technical Appendix. (0.05 MB DOC) [file pone.0012747.s001.doc]

**APPENDIX**

**The Cost-Effectiveness of Preventing AIDS Complications (CEPAC) International Model**

Model Structure

The CEPAC International Model is a widely-published, state transition model that simulates disease progression in a cohort of HIV-infected patients [1,2,3]. Patients enter the model individually and transition among three major health states: chronic disease, acute event, and death. Each patient enters the model in the chronic state and temporarily moves into an acute state with either an opportunistic infection or drug-related toxicity. Death can occur from either the chronic or acute state and can be caused by HIV-related complications, chronic disease, or non-AIDS causes. The model tracks each patient’s monthly clinical progression, recording the frequency of clinical events and resource utilization. At the time of death, the model counts clinical events, total life months, and accrued costs. Ten million patients are simulated in each analysis to ensure stable estimates of long-term survival and cost. Selected model input parameters, in addition to those in manuscript Table 1, Table 2, and Table 4, are in Table S1.

TB Mortality

All patients in this analysis enter the model with active TB. A patient with active TB disease is subject to both acute and extended mortality. Acute mortality is the probability of death during the first month after the onset of active TB. Extended mortality is the probability of death after the first month has passed. This mortality has three separate phases. Data for extended mortality are from a recent study of 1,088 smear-positive patients registered for TB treatment in 2002 or 2003 in a south Indian district who were followed for 2-3 years after completion of successful treatment (Table 2) [4].

**HIV Testing Offer/Acceptance and Outcomes**

Selective referral (Strategy 1) has a test offer/accept probability of 5.2% and detects 13.2% of those with HIV (Table 3) [5]. The test offer/accept probability was calculated by dividing the number of TB patients tested for HIV over the total number of TB patients registered for treatment with the RNTCP (77,000/1,475,587=5.2%) [5]. The proportion of HIV-TB co-infection detected was calculated by dividing the number of patients who tested positive for HIV-infection under the selective referral strategy (9,471 according to the RNTCP) over the total number of TB patients with HIV infection (the HIV prevalence among all TB patients times the total TB patients registered for treatment)—9,471/(4.85%*1,475,587)=13.2% [5].

The current standard (routine referral in the nine highest HIV prevalence states, with selective referral elsewhere; Strategy 2), has a test offer/accept probability of 22.7% and detects 44.7% of those with HIV. The test offer/accept probability is a weighted average of the test offer/accept probability for routine referral in the nine highest HIV prevalence states and the test offer/accept probability for selective referral in all other states.

The proportion of HIV-TB co-infection detected was calculated by dividing the number of patients who tested positive for HIV-infection under Strategy 2 over the total number of TB patients with HIV infection. To calculate the number of patients testing HIV-infected via Strategy 2, we first determined the number of patients tested for HIV under this strategy. We multiplied the HIV test offer/accept probability for routine referral (Strategy 3) by the total number of TB patients registered in the nine highest HIV prevalence states to find the number of patients tested in those states (66.2%*424,147=280,785) and added that number to the number of TB patients tested elsewhere (test offer/accept rate for selective referral times the number of TB patients registered in all other states—5.2%*1,051,440=54,675). To calculate the total number of TB patients testing HIV-positive under Strategy 2, we multiplied the numbers of patients getting tested from each group by the HIV prevalence in TB patients tested for HIV in those groups (9.0% in the high HIV prevalence states, and 12.3% elsewhere) [5,6]. We then divided that number by the total number of TB patients with HIV infection to get the proportion of HIV-TB co-infection detected (9%*280,785+12.3%*54,675)/(4.85%*1,475,587)=44.7%.

Routine referral for all TB patients (Strategy 3) has a test offer/accept probability of 66.2% and detects 66.2% of those with HIV [5,7]. The test offer/accept probability is from an RNTCP pilot study of provider-initiated referral of all TB patients for voluntary HIV testing and counseling [7]. The number of TB patients tested for HIV under Strategy 3 was the test offer/accept probability times the total number of registered TB patients (66.2%*1,475,587=976,839). The number of patients who tested positive for HIV-infection was the HIV prevalence among all TB patients times the number of TB patients tested for HIV under Strategy 3 (4.85%*976,839=47,377). The proportion of HIV-TB co-infection detected was calculated by dividing the number of patients who tested positive for HIV-infection under the routine referral strategy over the total number of TB patients with HIV infection (47,377/4.85%*1,475,587=66.2%).

**TB Treatment Outcomes**

Figure S1 shows the possible TB treatment outcomes for each of three groups of patients with regard to HIV status: HIV-negative, HIV-infected, but not tested and not treated for HIV, and HIV-infected, tested and treated for HIV. For each of these three HIV outcomes there are four TB outcomes: cured, failed TB treatment, defaulted (TB treatment interrupted for at least two consecutive months), or died. The projected mean life expectancy and costs from each of the 12 subgroups of TB and HIV patients are weighted to determine the overall projected mean life expectancy and costs for each alternative HIV testing strategy (Tables S2-S4).

**References**

1. Freedberg KA, Kumarasamy N, Losina E, Cecelia AJ, Scott CA, et al. (2007) Clinical impact and cost-effectiveness of antiretroviral therapy in India: starting criteria and second-line therapy. AIDS 21 Suppl 4: S117-128.

2. Goldie SJ, Yazdanpanah Y, Losina E, Weinstein MC, Anglaret X, et al. (2006) Cost-effectiveness of HIV treatment in resource-poor settings--the case of Côte d'Ivoire. N Engl J Med 355: 1141-1153.

3. Bender MA, Kumarasamy N, Mayer KH, Wang B, Walensky RP, et al. (2010) Cost-effectiveness of tenofovir as first-line antiretroviral therapy in India. Clin Infect Dis 50: 416-425.

4. Sadacharam K, Gopi PG, Chandrasekaran V, Eusuff SI, Subramani R, et al. (2007) Status of smear-positive TB patients at 2-3 years after initiation of treatment under a DOTS programme. Indian J Tuberc 54: 199-203.

5. RNTCP TB India 2008: RNTCP Status Report. In: Central TB Division DGoHS, editor. New Delhi.

6. Raizada N, Chauhan LS, Khera A, Sokhey J, Wares DF, et al. (2008) HIV seroprevalence among tuberculosis patients in India, 2006-2007. PLoS ONE 3: e2970.

7. Vijay S, Swaminathan S, Vaidyanathan P, Thomas A, Chauhan LS, et al. (2009) Feasibility of provider-initiated HIV testing and counselling of tuberculosis patients under the TB control programme in two districts of South India. PLoS ONE 4: e7899.

8. Swaminathan S, Deivanayagam CN, Rajasekaran S, Venkatesan P, Padmapriyadarsini C, et al. (2008) Long term follow up of HIV-infected patients with tuberculosis treated with 6-month intermittent short course chemotherapy. Natl Med J India 21: 3-8.

9. Mellors JW, Muñoz A, Giorgi JV, Margolick JB, Tassoni CJ, et al. (1997) Plasma viral load and CD4+ lymphocytes as prognostic markers of HIV-1 infection. Ann Intern Med 126: 946-954.

10. Losina E, Anglaret X, Yazdanpanah Y, Wang B, Touré S, et al. (2006) Impact of opportunistic diseases on chronic mortality in HIV-infected adults in Côte d'Ivoire. S Afr Med J 96: 526-529.

11. Yazdanpanah Y, Losina E, Anglaret X, Goldie SJ, Walensky RP, et al. (2005) Clinical impact and cost-effectiveness of co-trimoxazole prophylaxis in patients with HIV/AIDS in Cote d'Ivoire: a trial-based analysis. AIDS 19: 1299-1308.

12. Goldie SJ, Kaplan JE, Losina E, Weinstein MC, Paltiel AD, et al. (2002) Prophylaxis for human immunodeficiency virus-related *Pneumocystis carinii* pneumonia: using simulation modeling to inform clinical guidelines. Arch Intern Med 162: 921-928.

13. Management Sciences for Health (2009) The International Drug Price Indicator Guide.

14. Homan RK, Ganesh AK, Duraisamy P, Castle C, Sripriya M, et al. (2000) Economic Analyses of YRG CARE Services: implications for program sustainability. Research Triangle Park, NC, USA: Family Health International.

15. Cecelia AJ, Christybai P, Anand S, Jayakumar K, Gurunathan T, et al. (2006) Usefulness of an observational database to assess antiretroviral treatment trends in India. Natl Med J India 19: 14-17.

16. Ganesh AK Y. R. Gaitonde Centre for AIDS Research (YRG CARE) Pharmacy. Unit costs for components of HIV-related health care. Chennai, India.

17. Anglaret X, Chêne G, Attia A, Touré S, Lafont S, et al. (1999) Early chemoprophylaxis with trimethoprim-sulphamethoxazole for HIV-1-infected adults in Abidjan, Côte d'Ivoire: a randomised trial. Cotrimo-CI Study Group. Lancet 353: 1463-1468.

18. RNTCP TB India 2009: RNTCP Status Report. In: Central TB Division DGoHS, editor. New Delhi.
