# Supplementary material for: Cost-Effectiveness of HIV Testing Referral Strategies among Tuberculosis Patients in India
Source: PLoS One. 2010 Sep 16;5(9):e12747. doi: 10.1371/journal.pone.0012747 (PMC2940842; doi:10.1371/journal.pone.0012747)
Supplement: Table S1 — Baseline cohort characteristics and model inputs for an analysis of HIV testing for TB patients in India. (0.10 MB DOC) [file pone.0012747.s002.doc]

**Table S1. Baseline cohort characteristics and model inputs for an analysis of HIV testing for TB patients in India.**

| **Parameter** | **Base case input** | **Reference** |
| --- | --- | --- |
| **Natural History** |  |  |
| Distribution of initial HIV RNA, copies/ml |  | [8] |
| >100,000 | 0.590 |  |
| 30,001-100,000 | 0.180 |  |
| 10,001-30,000 | 0.094 |  |
| 3001-10,000 | 0.049 |  |
| 501-3000 | 0.025 |  |
| 0-500 | 0.062 |  |
| Mean monthly CD4 cell decline (cells/µl), |  | [9] |
| stratified by HIV RNA level (copies/ml) |  |  |
| >30,000 | 6.4 |  |
| 10,001-30,000 | 5.4 |  |
| 3001-10,000 | 4.6 |  |
| 501-3000 | 3.7 |  |
| 0-500 | 3.0 |  |
| CD4-dependent incidence of mild opportunistic infections, monthly (%) |  | [1] |
| Mild bacterial infection | 0.00-0.50 |  |
| Mild fungal infection | 0.32-8.12 |  |
| Other mild infection | 0.56-2.71 |  |
| **Natural History** |  |  |
| CD4-dependent incidence of severe opportunistic infections, monthly (%) |  | [1] |
| Tuberculosis | 0.23-5.95 |  |
| Malaria | 0.00-0.02 |  |
| Visceral Stage 3-4 | 0.20-1.37 |  |
| Non-visceral Stage 3-4 | 0.27-1.05 |  |
| Non-specific Stage 3-4 | 0.11-2.29 |  |
| Severe bacterial infection | 0.00-0.22 |  |
| Other severe infection | 0.23-2.65 |  |
| Chronic, CD4 count-dependent AIDS-related mortality, monthly risk (%) |  | [10] |
| Without prior opportunistic infection | 0.04-2.10 |  |
| With prior opportunistic infection | 0.98-5.40 |  |
| **Efficacy of co-trimoxazole (% reduction in probability of infection)** |  | [11,12] |
| Mild bacterial infection | 48.8 |  |
| Severe bacterial infection | 49.8 |  |
| Mild fungal infection* | -46.4 |  |
| Malaria | 88.4 |  |
| Other severe infections | 17.9 |  |
| **Costs (2008 US$)** |  |  |
| Drug-related toxicities |  | [11,14] |
| Minor toxicity | 16 |  |
| Major toxicity | 174 |  |
| Opportunistic infection treatment |  | [14] |
| Mild bacterial infection | 25 |  |
| Severe bacterial infection | 59 |  |
| Visceral Stage 3-4 | 82 |  |
| Non-visceral Stage 3-4 | 32 |  |
| Non-specific Stage 3-4 | 35 |  |
| Malaria | 142 |  |
| Mild fungal infection | 32 |  |
| Other mild infection | 23 |  |
| Other severe infection | 34 |  |
| Terminal care (last month of life) | 12 | [14,15] |
| HIV clinic visit | 15 | [14] |
| Hospital day | 23 | [14] |
| CD4 test | 5 | [16] |

*Mild fungal infections increased by co-trimoxazole [11,17].
