# Supplementary material for: Cost-Effectiveness of HIV Testing Referral Strategies among Tuberculosis Patients in India
Source: PLoS One. 2010 Sep 16;5(9):e12747. doi: 10.1371/journal.pone.0012747 (PMC2940842; doi:10.1371/journal.pone.0012747)
Supplement: Table S2 — Weighted cost and life expectancy by TB outcomes. (0.05 MB DOC) [file pone.0012747.s003.doc]

**Table S2.** **Weighted cost and life expectancy by TB outcomes.**

| **Strategy** | |  | **Cured** | | |  | | **Defaulted** | | |  | | **Died** | | |  | | **Failed** | | |  | |
| --- | --- | --- | --- | --- | --- | --- | --- | --- | --- | --- | --- | --- | --- | --- | --- | --- | --- | --- | --- | --- | --- | --- |
|  |  | % in category* | | Life Months | Cost ($) | | % in category | | Life Months | Cost ($) | | % in category | | Life Months | Cost ($) | | % in category | | Life Months | Cost ($) | |  |
| Strategy 1: Selective referral | |  | |  |  | |  | |  |  | |  | |  |  | |  | |  |  | |  |
|  | HIV detected (13%) |  | |  |  | |  | |  |  | |  | |  |  | |  | |  |  | |  |
|  | TB, HIV - | 83.0 | | 236.8 | 55 | | 8.3 | | 130.3 | 15 | | 6.3 | | 4.5 | 40 | | 2.4 | | 152.3 | 40 | |  |
|  | TB, HIV +, untreated | 61.0 | | 44.4 | 1,410 | | 14.0 | | 13.4 | 315 | | 22.0 | | 4.2 | 50 | | 3.0 | | 24.7 | 730 | |  |
|  | TB, HIV +, treated | 61.0 | | 122.7 | 5,390 | | 14.0 | | 19.7 | 725 | | 22.0 | | 4.3 | 130 | | 3.0 | | 63.5 | 2,690 | |  |
| Strategy 2: Routine referral in 9 states, high-risk in other states | | | | | | |  | |  |  | |  | |  |  | |  | |  |  | |  |
|  | HIV detected (45%) |  | |  |  | |  | |  |  | |  | |  |  | |  | |  |  | |  |
|  | TB, HIV - | 83.0 | | 236.8 | 55 | | 8.3 | | 130.3 | 15 | | 6.3 | | 4.5 | 40 | | 2.4 | | 152.3 | 40 | |  |
|  | TB, HIV +, untreated | 61.0 | | 44.4 | 1,410 | | 14.0 | | 13.4 | 315 | | 22.0 | | 4.2 | 50 | | 3.0 | | 24.7 | 730 | |  |
|  | TB, HIV +, treated | 61.0 | | 122.7 | 5,390 | | 14.0 | | 19.7 | 725 | | 22.0 | | 4.3 | 130 | | 3.0 | | 63.5 | 2,690 | |  |
| Strategy 3: Routine referral in all states | | | |  |  | |  | |  |  | |  | |  |  | |  | |  |  | |  |
|  | HIV detected (66%) |  | |  |  | |  | |  |  | |  | |  |  | |  | |  |  | |  |
|  | TB, HIV - | 83.0 | | 236.8 | 55 | | 8.3 | | 130.3 | 15 | | 6.3 | | 4.5 | 40 | | 2.4 | | 152.3 | 40 | |  |
|  | TB, HIV +, untreated | 61.0 | | 44.4 | 1,410 | | 14.0 | | 13.4 | 315 | | 22.0 | | 4.2 | 50 | | 3.0 | | 24.7 | 730 | |  |
|  | TB, HIV +, treated | 61.0 | | 122.7 | 5,390 | | 14.0 | | 19.7 | 725 | | 22.0 | | 4.3 | 130 | | 3.0 | | 63.5 | 2,690 | |  |

HIV-: HIV-negative; HIV+: HIV-infected. Base case HIV prevalence = 4.9% [18].

*Each row across for % in category adds to 100%.
