# Supplementary material for: Cost-Effectiveness of HIV Testing Referral Strategies among Tuberculosis Patients in India
Source: PLoS One. 2010 Sep 16;5(9):e12747. doi: 10.1371/journal.pone.0012747 (PMC2940842; doi:10.1371/journal.pone.0012747)
Supplement: Table S3 — Weighted cost and life expectancy by TB outcomes (continued). (0.05 MB DOC) [file pone.0012747.s004.doc]

**Table S3.** **Weighted cost and life expectancy by TB outcomes (continued).**

| **Strategy** | **Weighted Life Months** | **Weighted Cost ($)** | **% in Category, Perfect Linkage** | **Linkage to HIV Care (%)** | **% in Category, Actual Linkage** | **Total Life Months** | **Total Cost ($)** |
| --- | --- | --- | --- | --- | --- | --- | --- |
| Strategy 1: Selective referral | |  |  |  |  |  |  |
| TB, HIV - | 211.3 | 50 | 95.2 | n/a | 95.2 | 201.0 | 50 |
| TB, HIV +, untreated | 30.6 | 935 | 4.2 | 26 | 4.7 | 1.4 | 45 |
| TB, HIV +, treated | 80.4 | 3,500 | 0.6 | 26 | 0.2 | 0.1 | 5 |
| Weighted total* |  |  | 100.0 |  | 100.0 | 202.6 | 100 |
| Strategy 2: Routine referral in 9 states, high-risk in other states | | | |  |  |  |  |
| TB, HIV - | 211.3 | 50 | 95.2 | n/a | 95.2 | 201.0 | 50 |
| TB, HIV +, untreated | 30.6 | 935 | 2.7 | 26 | 4.3 | 1.3 | 40 |
| TB, HIV +, treated | 80.4 | 3,500 | 2.2 | 26 | 0.6 | 0.5 | 20 |
| Weighted total* |  |  | 100.0 |  | 100.0 | 202.8 | 110 |
| Strategy 3: Routine referral in all states | |  |  |  |  |  |  |
| TB, HIV - | 211.3 | 50 | 95.2 | n/a | 95.2 | 201.0 | 50 |
| TB, HIV +, untreated | 30.6 | 935 | 1.6 | 26 | 4.0 | 1.2 | 40 |
| TB, HIV +, treated | 80.4 | 3,500 | 3.2 | 26 | 0.8 | 0.7 | 30 |
| Weighted total* |  |  | 100.0 |  | 100.0 | 202.9 | 120 |

HIV-: HIV-negative; HIV+: HIV-infected. Base case HIV prevalence = 4.9% [18].

*Weighted totals include $3 cost for those tested for HIV. Totals may not equal 100% due to rounding.
