# Supplementary material for: Cost-Effectiveness of HIV Testing Referral Strategies among Tuberculosis Patients in India
Source: PLoS One. 2010 Sep 16;5(9):e12747. doi: 10.1371/journal.pone.0012747 (PMC2940842; doi:10.1371/journal.pone.0012747)
Supplement: Table S4 — Weighted cost and life expectancy by TB outcomes (continued). (0.03 MB DOC) [file pone.0012747.s005.doc]

**Table S4. Weighted cost and life expectancy by TB outcomes (continued).**

| **Strategy** | **Life Months** | **Cost ($)** | **Incremental Cost-Effectiveness Ratio ($/YLS)** |
| --- | --- | --- | --- |
| Strategy 1 | 202.6 | 100 | -- |
| Strategy 2 | 202.8 | 110 | 650 |
| Strategy 3 | 202.9 | 120 | 730 |
