# Supplementary figures and images for: Cost-Effectiveness of HIV Testing Referral Strategies among Tuberculosis Patients in India
Source: PLoS One. 2010 Sep 16;5(9):e12747. doi: 10.1371/journal.pone.0012747 (PMC2940842; doi:10.1371/journal.pone.0012747)

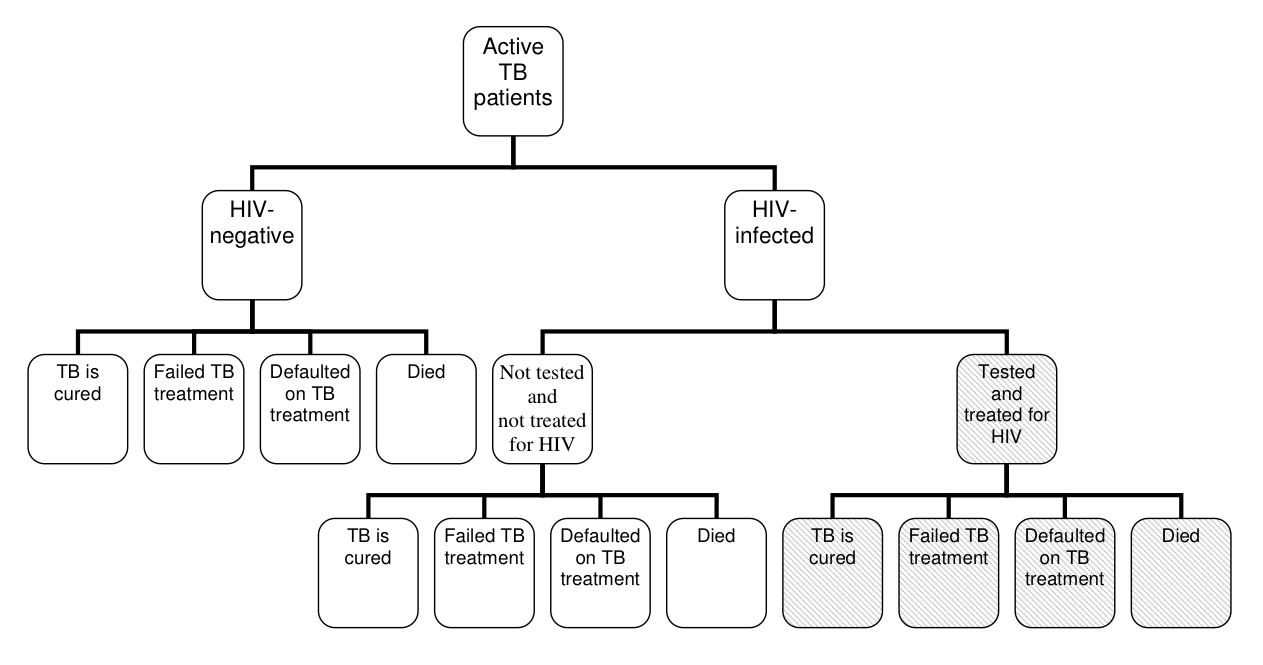

Supplement: Figure S1 — Twelve patient subgroups in a model of HIV testing among TB patients in India. Grey shading indicates those patients tested and treated for HIV infection. (2.52 MB TIF) [file pone.0012747.s006.tif]
